# Psychometric properties of the Chinese version of the Attitudes Toward Accompanied Driving Scale and its relationship with driving styles

**DOI:** 10.1371/journal.pone.0242374

**Published:** 2020-11-19

**Authors:** Long Sun, Yueying Pan, Ye Tian

**Affiliations:** School of Psychology, Liaoning Normal University, Dalian, Liaoning, P. R. China; Universitat de Valencia, SPAIN

## Abstract

The present study aimed to adapt the Attitudes Toward Accompanied Driving Scale (ATADS) to a Chinese drivers sample and to examine its reliability and validity. Five hundred and seventy-two drivers aged 18 to 25 years old were asked to complete the ATADS and a validated Chinese version of the Multidimensional Driving Style Inventory. The factorial structure of the ATADS was examined using exploratory factor analysis (*N* = 259) and confirmatory factor analysis (*N* = 313). The validity of the scale was evaluated by examining the associations between the ATADS factors, demographic variables and driving styles. The results showed that both the findings of the EFA and CFA showed a five-factor structure of the ATADS, including tension, relatedness, avoidance, disapproval and anxiety. Second, significant gender differences were found in tension, relatedness, avoidance and anxiety. Third, tension, avoidance, disapproval and anxiety were moderately or weakly correlated with risky, anxious, angry and careful driving styles. Moreover, the number of traffic accidents after the accompanying phase was positively correlated with disapproval and avoidance. The findings supported the psychometric properties of the Chinese version of the ATADS and highlighted the adverse effects of young drivers’ negative attitudes toward accompanied driving on their driving styles.

## Introduction

Young novice drivers are found to have the highest crash risk among all drivers. Their crash rate is highest during the first month of driving after they obtain their driving license, after which it declines rapidly for approximately six months, and then it declines more slowly in the following two years [1,2]. In China, the Nanjing Traffic Management Department has analyzed big data on traffic accidents caused by young novice drivers and has found that the accident rate of young novice drivers with less than one year of driving experience is more than 4 times that of drivers with over 10 years of experience [3]. Although many human factors accounted for traffic accidents on the road, most studies in transport psychology focus on young drivers’ personality characteristics [4,5]. Despite personality characteristics, studies have found that young drivers’ attitudes toward accompanied driving also play important roles in their driving safety [6,7]. Accompanied driving refers to young drivers being legally required to drive with a parent or other experienced driver following licensure. To measure young drivers’ attitudes towards accompanying driving, a 23-item scale called Attitudes Toward Accompanied Driving Scale (ATADS) was developed in Israel in 2010 [6]. Due to lack of instruments in assessing young drivers’ attitudes towards accompanied driving in China, the present study aimed to adapt the ATADS to Chinese language and examine its reliability and validity.

To justify the adaptation and validation of the ATADS, linguistic, psychological and cultural differences between the populations of China and Israel were taken into consideration in line with the guidelines of International Test Commission [8]. According to a recent global status report on road safety released by World Health Organization [9], the number of road traffic deaths per 10, 0000 populations was 4.1 in China in 2015 and 4.4 in Israel in 2016. The seatbelt wearing rates in front seats in China was 37% while in Israel it was 89%. Additionally, the road deaths caused by the consumption of alcohol were less than 1% in China while in Israel it was about 4%. In China, accompanied driving is only required when young teens drive on the high-speed way after they obtain driving licenses. The age to obtain a driving license is at least 18 years old. However, teens can begin taking driving lessons at the age of 16.5 and accompanied driving is a compulsory step during the driving of the first three months In Israel.

The ATADS includes five factors, namely, tension, relatedness, avoidance, disapproval and anxiety. Tension refers to the tendency to perceive the experience of being accompanied as distressing and angry. Relatedness refers to the tendency to perceive accompanied driving as an opportunity to get closer to the accompanying driver. Avoidance refers to the tendency to spend as little time as possible to avoid accompanying driving. Disapproval refers to the tendency to receive and express criticism and disapproval during accompanied driving. Anxiety refers to the tendency to be afraid of being involved in accidents or making mistakes in front of the accompanying driver. The reliabilities of the ATADS factors were satisfactory in Israel samples [6,10].

### Attitudes toward accompanied driving and traffic violations

Since its publication in 2010, the ATADS has been used to describe and understand the accompanied driving period and the associations between attitudes toward accompanied driving and traffic accidents in Israel [6,10,11]. One study found that young drivers rate themselves as less careful and courteous drivers if they score higher on negative attitudes toward accompanied driving [12]. Another study found that young drivers who score higher on anxiety and lower on disapproval are more likely to see reckless driving as being risky [6]. Additionally, compared to drivers who do not favor accompanied driving, young drivers who receive more accompanied driving are less likely to be involved in car crashes [13]. Although previous studies have not directly examined the relationship between young drivers’ traffic accidents and their attitudes toward accompanied driving, these findings have highlighted that their attitudes do affect their involvement in traffic accidents immediately after the accompanied driving period.

### Attitudes toward accompanied driving and driving styles

Despite the associations between young drivers’ attitudes toward accompanied driving and traffic accidents, another important issue that needs to be solved is whether their attitudes affect their driving behaviors or driving style. Driving style refers to the ways that individuals choose to habitually drive [14]. Many factors affect an individual’s driving style, among which their family’s attitude toward road safety and their significant role models are two key factors [15]. Given that young drivers’ main accompanied drivers are usually their parents [16], young drivers’ attitudes are found to be associated with their parents’ driving style. For example, young drivers’ negative attitudes, such as disapproval and avoidance, are positively associated with their parents’ maladaptive driving styles, such as risky driving style [11]. More directly, one study found that young male drivers’ negative attitudes, such as disapproval, are positively associated with their frequency of reckless driving [6]. The findings indicate that young drivers’ negative attitudes toward accompanied driving might result in risky driving styles. Therefore, the present study will examine the effect of attitude toward accompanied driving on driving style.

### Attitudes toward accompanied driving and demographic characteristics

Demographic factors also affected young drivers’ attitude toward accompanied driving. However, mixed results have been reported regarding the associations between demographic factors and ATADS factors. The Israel study found that male drivers score higher on tension, avoidance and anxiety than do female drivers [6]. However, studies have also shown that female drivers tend to exhibit more driving stress than do male drivers [17,18] and that female drivers have more distracted driving behaviors than do male drivers [19]. On the other hand, positive correlations between age and relatedness have been found [6,10]. One study found that age is positively correlated with tension [6], while other studies have found either a negative correlation [11] or no significant correlation [10]. Furthermore, to our knowledge, no studies have examined the effects of years of education on young drivers’ attitudes toward accompanied driving. Therefore, the present study will further examine the relationships between demographic factors and ATADS factors using a sample of Chinese drivers.

### Aims of the present study

The main purpose of the present study is to adapt and validate the ATADS in Chinese culture. The second purpose is to determine whether ATADS factors affect the establishment of young drivers’ driving styles, thus helping develop some interventions or training programs in China.

## Method

### Participants

This study was approved by the Logistics Department for Civilian Ethics Committee of Liaoning Normal University. Six hundred drivers with accompanied driving experience were recruited from Dalian, Shanghai, Xi’an and Kunming. Participants received a gift coupon (15 yuan RMB) upon answering all of the questions. The participants had held a driving license for 3–36 months, ranging in age from 18 to 25. After examining all the collected data, the data of 28 participants were determined to be invalid due to the absence of some answers and were thus discarded. This study included two samples for exploratory factor analysis and confirmatory factor analysis. Sample one consisted of 100 male drivers (38.6%) and 159 female drivers (61.4%). A total of 18.5% of the participants had a middle school education, 33.2% had a high school education, and 48.3% had a college education. With regard to the accompanying drivers, 55.6% of the participants were accompanied by their father, 28.2% were accompanied by their mother and 16.2% were accompanied by another family member or a peer. Table 1 shows detailed socio-demographic information.

**Table 1:** Socio-demographic information of participants (sample one).

Socio-demographic information	Still on accompanied driving (*N* = 183)				After the accompanied driving phase (*N* = 76)			
	Min	Max	Mean	SD	Min	Max	Mean	SD
Age(yr)	18	25	20.55	1.54	20	25	23.36	1.28
Gender ^a^	1	2	1.75	0.44	1	2	1.29	0.46
Years of experience (yr)	0.25	1	0.91	0.20	1.5	3	2.36	0.56
Years of education (yr)	9	16	14.13	2.56	9	16	11.55	2.29
Driving frequency ^b^	1	4	1.67	0.84	1	3	1.57	0.60
Main accompanied driver ^c^	1	3	2.42	0.83	1	3	1.92	0.88
Hours of accompanied driving	3	30	9.69	3.96	15	30	23.55	5.59
Number of violations	0	5	0.22	0.78	0	7	0.96	1.42
Number of crashes	0	1	0.02	0.13	0	3	0.21	0.52

Note:

^a^ 1 = Male, 2 = Female.

^b^ 1 = daily, 2 = weekly, 3 = monthly, 4 = rarely

^c^ 1 = Father, 2 = Mother, 3 = Another family member or a peer

Sample two consisted of 125 male drivers (39.9%) and 188 female drivers (60.1%). A total of 14.7% of participants had a middle school education, 30.7% had a high school education, and 54.6% had a college education. With regard to the accompanying drivers, 53% were accompanied by their father, 27.8% were accompanied by their mother and 19.2% were accompanied by another family member or a peer. Table 2 shows detailed socio-demographic information.

**Table 2:** Socio-demographic information of participants (sample two).

Socio-demographic information	Still on accompanied driving (*N* = 233)				After the accompanied driving phase(*N* = 80)			
	Min	Max	Mean	SD	Min	Max	Mean	SD
Age(yr)	18	25	20.41	1.92	18	25	20.67	1.78
Gender ^a^	1	2	1.64	0.48	1	2	1.49	0.50
Years of experience (yr)	0.25	1	0.77	0.26	1	2.5	1.78	0.36
Years of education (yr)	9	16	13.89	2.58	9	16	13.31	2.84
Driving frequency ^b^	1	4	1.95	0.80	1	3	1.71	0.67
Main accompanied driver ^c^	1	3	1.68	0.81	1	3	1.61	0.68
Hours of accompanied driving	2.5	15	7.91	3.03	15	35	19.21	5.39
Number of violations	0	3	0.15	0.44	0	6	0.43	0.96
Number of crashes	0	1	0.06	0.23	0	3	0.16	0.49

Note:

^a^ 1 = Male, 2 = Female.

^b^ 1 = daily, 2 = weekly, 3 = monthly, 4 = rarely

^c^ 1 = Father, 2 = Mother, 3 = Another family member or a peer

### Measures

#### Attitudes Toward Accompanied Driving Scale

The original ATADS includes 23 items with satisfactory reliabilities. The scale contained 5 factors: tension (10 items), relatedness (2 items), avoidance (3 items), disapproval (4 items) and anxiety (4 items) [6]. Participants are asked to read each of the items and rate the extent to which it fits their feelings, thoughts, and behaviors during the accompanied driving period on a 5-point Likert scale ranging from 1 (not at all) to 5 (very much).

#### Multidimensional Driving Style Inventory-Chinese version (MDSI-C)

The MDSI-C contained 32 items and could assess four driving styles called risky, angry, careful and anxious driving style [20]. Risky driving style (6 items, *α* = 0.80), refers to sensation seeking and reckless driving. Angry driving style (6 items, *α* = 0.82), refers to the tendency to experience irritation, rage and drive aggressively. Careful driving style (6 items, *α* = 0.89), refers to careful and safe driving behaviors. Anxious driving style (14 items, *α* = 0.76), refers to the feelings of distress and tension, as well as distracted activities during driving. Participants were asked to rate each item on a 6-point Likert scale, ranging from 1 (not at all) to 6 (very much).

#### Procedure

The original ATADS was first translated into Chinese upon the permission of the right holder of the scale. Following the translation/back-translation procedure, the ATADS was translated by an independent Chinese researcher and this version was then translated into English by a bilingual Chinese and English researcher. After the differences in the translations were properly solved, an initial 23-item scale was got.

Next, the ATADS and MDSI-C were sent to four postgraduate students whose families were located in Dalian, Shanghai, Xi’an and Kunming. The students sent out the scales to their friends via the internet for the first round of data collection from April 1–7, 2019 and for the second round of data collection from May 1–7, 2019. Only those participants who had accompanied driving experiences were provided with an informed consent form and they were asked to finish the scales within 30 minutes. Participants were also required to provide demographic information, including sex, age, average number of hours of accompanied driving, main accompanied driver and number of traffic violations after the accompanied driving phase.

### Statistical analysis

The data were analyzed in four steps using SPSS version 18.0. This study first conducted EFA and CFA to explore the factorial structure of the ATADS. As previous study recommended [21], the cut-off values in the CFA were set at .90 for the goodness-of-fit index (GFI) and Turke-Lewis index (TLI), .08 for the root mean square error of approximation (RMSEA), and at 5 for the ratio of the chi-square to its degrees of freedom(*X*^*2*^*/df*). Pearson correlations between the ATADS-C factors and driving styles were analyzed to explore its validity. Given that demographic characteristics are important in investigations of factors such as attitudes and behaviors [6,10], univariate ANOVAs were conducted to examine the effects of sex and main accompanied driver and Pearson correlations were conducted to examine the relationships between the ATADS-C factors and age, average number of hours of accompanied driving, as well as traffic violations. The predictive roles of the ATADS-C factors on driving styles were examined using hierarchical regression analyses.

## Results

### Factor analysis

The 23 items were subjected to an EFA using primary axis extraction and varimax rotation. The first analysis revealed 6 factors that explained 62.39% of the total variance. Three items generated a new factor with the Cronbach’s alpha lower than 0.70, and hence were discarded. The results of the second EFA revealed 5 factors that explained 60.74% of the total variance. The five factors were named as tension, disapproval, anxiety, avoidance and relatedness, which can explain 17.39%, 13.66%, 10.98%, 9.62% and 9.1% of the variance, respectively. The factor loadings are shown in Table 3.

**Table 3:** Psychological property of the ATADS.

Items	M	SD	Median	Skewness	Kurtosis	Factor loading
*Tension*						
3.	2.64	1.08	3	0.03	-0.92	0.72
7.	2.27	1.01	2	0.21	-0.88	0.68
4.	2.86	1.01	3	0.03	-0.42	0.64
1.	2.50	1.08	3	0.09	-0.93	0.62
6.	1.97	0.97	2	0.79	-0.01	0.62
2.	1.83	0.91	2	0.99	0.55	0.60
5.	2.17	0.93	2	0.41	0.33	0.58
*Disapproval*						
12.	2.00	0.91	2	0.69	0.04	0.76
11.	2.19	0.92	2	0.41	-0.25	0.73
10.	2.20	1.05	2	0.59	0.36	0.74
13.	2.31	0.94	2	0.33	-0.38	0.48
*Anxiety*						
15.	3.14	1.13	3	-0.46	-0.71	0.71
17.	2.30	1.00	2	0.19	-0.96	0.69
16.	2.27	1.04	2	0.43	-0.62	0.65
14.	2.78	1.21	3	-0.19	-1.22	0.60
*Avoidance*						
18.	2.73	1.03	3	0.03	-0.65	0.62
19.	2.72	1.06	3	-0.02	-0.67	0.70
20.	2.71	1.04	3	0.02	-0.83	0.77
*Relatedness*						
8.	2.92	1.03	3	-0.10	-0.33	0.91
9.	2.86	0.99	3	-0.15	-0.31	0.93

**Fig 1:**
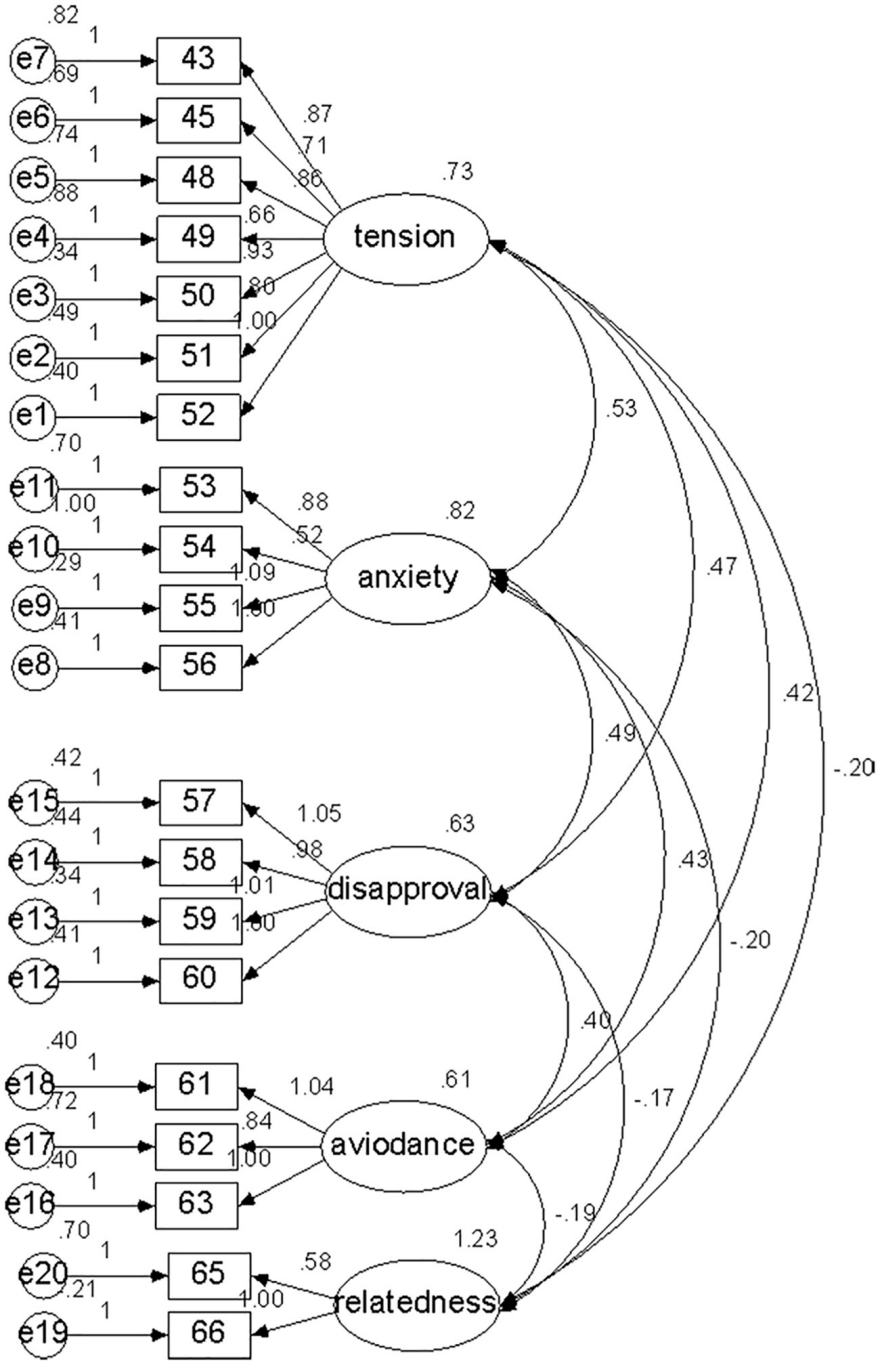
Path diagram.

A CFA was further performed using maximum likelihood estimation method to examine the model fitness of the ATADS-C via AMOS version 17.0. The results showed that the indices of the model fit were acceptable (*X*^*2*^ = 346.97, *df* = 157, *X*^*2*^*/df* = 2.21, GFI = 0.90, TLI = 0.92). The path diagram is shown in Fig 1.

### Internal consistency of the ATADS-C

Reliability analyses showed the Cronbach’s alpha coefficient of the whole scale was 0.86. The Cronbach’s alpha coefficient of the sub-factors was 0.818 for tension, 0.775 for disapproval, 0.747 for anxiety, 0.618 for avoidance and 0.855 for relatedness. Despite of avoidance, the reliabilities of the other four factors were acceptable.

### Validity of the ATADS-C

The discriminant validity of the ATADS-C was supported by the significant associations, for the most part, found between the factors of the ATADS-C and the MDSI-C factors (see Table 4).

**Table 4:** Correlation between the ATADS-C factors and driving style.

ATADS-C factors	Tension	Disapproval	Anxiety	Avoidance	Relatedness
Disapproval	0.53**				
Anxiety	0.55**	0.50**			
Avoidance	0.39**	0.45**	0.42**		
Relatedness	-0.14*	-0.14*	-0.06	0.02	
Anxious style	0.47**	0.34**	0.46**	0.37**	0.02
Risky style	0.19**	0.29**	0.06	0.14*	-0.21**
Angry style	0.26**	0.36**	0.17*	0.18**	-0.08
Careful style	-0.20**	-0.17*	-0.03	0.01	0.21**

Note:

*p< .05,

**p< .01.

Tension, disapproval and avoidance were positively and significantly correlated with angry, risky and anxious driving styles. Anxiety was positively and significantly correlated with anxious and angry driving styles. Tension and disapproval were negatively and significantly correlated with careful driving style. Relatedness was positively and significantly correlated with careful driving style and negatively correlated with risky driving style.

### Effects of sex

One-way ANOVAs (see Table 5) showed that male drivers had lower scores on tension, anxiety, avoidance and relatedness than did female drivers. No sex differences were found on the score of disapproval.

**Table 5:** Means of the ATADS-C factors by sex.

ATADS-C factors	Sex	*N*	Mean	SD	*F*(1,257)	Cohen’s *d*
Tension	Male	100	2.21	0.74	4.20*	-0.26
	Female	159	2.39	0.66		
Disapproval	Male	100	2.25	0.86	1.65	0.18
	Female	159	2.13	0.65		
Anxiety	Male	100	2.39	0.83	13.16**	-0.47
	Female	159	2.77	0.79		
Avoidance	Male	100	2.58	0.84	5.00*	-0.29
	Female	159	2.81	0.73		
Relatedness	Male	100	2.74	0.99	3.65*	-0.24
	Female	159	2.97	0.90		

Note:

*p< .05,

**p< .01.

### Effects of main accompanied drivers

One-way ANOVA also showed that the effect of the identity of the main accompanied drivers did not significantly affect young drivers’ attitudes toward accompanied driving, *F*(10, 506) = 1.49, *p* = .14.

### ATADS-C factors and age and hours of accompanied driving

Pearson correlations revealed that age was negatively correlated with tension (*r* = -0.22, *p*< .01), anxiety (*r* = -0.14, *p*< .05) and relatedness (*r* = -0.14, *p*< .05). The average number of hours of accompanied driving was negatively correlated with tension (*r* = -0.32, *p*< 0.01), disapproval (*r* = -0.16, *p*< .05), anxiety (*r* = -0.32, *p*< .01) and avoidance (*r* = -0.28, *p*< .01). Years of education were positively correlated with tension (*r* = 0.29, *p*< .01), disapproval (*r* = 0.15, *p*< .05), anxiety (*r* = 0.37, *p*< .01) and avoidance (*r* = 0.32, *p*< .01). These results suggest that the more hours of accompanied driving and the fewer years of education young drivers receive, the less likely they are to show a negative attitude toward accompanied driving.

### ATADS-C factors and traffic violations

The number of traffic violations after the accompanied driving phase was positively correlated with anxiety (*r* = 0.32, *p*< .01) and disapproval (*r* = 0.38, *p*< .01). Similarly, the number of traffic accidents was positively correlated with disapproval (*r* = 0.30, *p*< .01) and avoidance (*r* = 0.30, *p*< .01). These results suggest that young drivers who disapprove or avoid accompanied driving have more traffic accidents.

### Prediction of the ATADS-C factors on driving styles

The predictions of the ATADS-C factors on driving styles were further examined using hierarchical regression analyses (enter method). The results are showed in Table 6.

**Table 6:** Hierarchical regression coefficients for the prediction of driving styles.

Variables	Anxious style			Risky style			Angry style			Careful style		
	*β*	*SE*	*t*	*β*	*SE*	*t*	*β*	*SE*	*t*	*β*	*SE*	*t*
Tension	0.27	0.07	4.05**	0.08	0.07	1.08	0.11	0.10	1.50	-0.21	0.11	-2.69**
Disapproval	0.02	0.06	0.25	0.28	0.07	3.75**	0.32	0.09	4.29**	-0.14	0.10	-1.86
Anxiety	0.25	0.05	3.67**	-0.16	0.06	-2.07*	-0.07	0.08	0.94	0.13	0.09	1.70
Avoidance	0.15	0.05	2.47*	0.05	0.06	0.77	0.03	0.08	0.40	0.09	0.09	1.31
Relatedness	0.07	0.04	1.38	-0.17	0.04	2.81**	-0.02	0.06	-0.35	0.17	0.06	2.78**
*F*	22.113**			7.606**			8.208**			5.535**		
*ΔR*^*2*^	0.290			0.113			0.123			0.081		

Note:

*p< .05,

**p< .01.

Table 6 showed that for anxious driving style, tension, anxiety and avoidance could predict it and explained 29% of the variance in this style. For angry driving style, disapproval could predict it and explained 12.3% of the variance in this style. For risky driving style, disapproval, anxiety and relatedness could predict it and explained 11.3% of the variance in this style. And for careful driving style, tension and relatedness could predict it and explained 8.1% of the variance in this style.

## Discussion

The purpose of the present study is to provide a reliable and valid instrument to assess young drivers’ attitudes toward accompanied driving in China. To achieve this goal, we adapted the ATADS to a Chinese drivers sample and examined its associations with driving styles and traffic violations.

First, the findings of EFA and CFA showed the factorial structural of the ATADS was similar to the results obtained in the Israel sample [6]. The final version of the ATADS-C contained 20 items, which can be divided into 5 factors, namely, tension, disapproval, anxiety, avoidance and relatedness. The present study found that tension, disapproval, anxiety, and avoidance were positively correlated with each other. Relatedness was only negatively correlated with tension and disapproval. However, contrary to the findings of an Israel study [6], no significant correlation was found between relatedness and avoidance in this study.

Second, positive and significant correlations were found between anxious, risky and angry driving styles and tension, disapproval and avoidance. These results were supported by the findings of an Israel study showing that young drivers’ scores on tension, disapproval, anxiety and avoidance correlate positively with their parents’ anxious, risky and angry driving styles [10]. This study found that young drivers who express criticism toward their principal accompanied driver tend to adopt a risky or angry driving style. This result is consistent with the finding of an Israel study where disapproval can predict young male drivers’ increased frequency of reckless driving [6]. The results also indicate that drivers who score higher on disapproval might experience more traffic accidents after the accompanying driving phase [20]. It is understandable that young drivers who are against accompanied driving might be overconfident about their driving ability, or they could get angry easily because they might see accompanied driving as a burden or damaging to their self-esteem.

In line with the findings of previous studies [14,22], the present study found that tension and anxiety were good predictors of anxious driving style. One possible explanation is that young drivers who are striving for the control of the vehicle might see accompanied driving as a threat to their freedom. Thus, they may experience more pressure or feel frustrated during accompanied driving. This, in turn, might result in a feeling of distress or distracted driving behaviors [14]. Noticeably, avoidance can also predict anxious driving style. This outcome is supported by the findings of a previous study where parents’ anxious driving style was positively associated with young drivers’ avoidance [10]. The result suggested that young drivers avoid accompanied driving because of their failure to reduce anxiety while driving.

Furthermore, young drivers who scored lower on tension and higher on relatedness were more likely to adopt careful driving style. This outcome is supported by a previous study showing that positive family dynamics and high adaptation can reduce young drivers’ tension and anxiety during accompanied driving [11]. Another study also found that parents’ higher involvement in accompanied driving, such as modeling safe driving and giving feedback to their offspring’s driving in varying situations, is positively associated with their offspring’s careful driving style [16]. China is a strongly collectivistic cultural country in which people have many more interactions and shared beliefs among family members [23]. Thus, young Chinese drivers feel much closer with their parents, which in turn might help reduce their tension and make them feel safer while driving.

Finally, this study found that female drivers scored higher on tension, anxiety and avoidance than male drivers, suggesting that female drivers are more likely to feel anxious during accompanied driving and that they also tend to avoid accompanied driving. These findings are consistent with the findings of previous studies showing that female drivers tend to exhibit more driving stress than do male drivers [17,18,24]. Negative correlations were found between age, tension, anxiety and relatedness. However, an Israel study found that age is positively correlated with relatedness and avoidance [6]. Notably, despite the possible influence of social culture [23], the age (at least 18 years old) of Chinese teens to obtain driving license is relatively greater than that of Israeli drivers, which might reduce their tendency to feel distressed in driving [20]. This was supported by the findings of other studies that the older a driver is, the less he or she will experience anxiety in driving [14,20]. The present study is the first to find that young drivers’ negative attitudes are negatively correlated with their average number of hours of accompanied driving and positively correlated with years of education. These results suggest that the more hours of accompanied driving and fewer years of education young drivers receive, the less likely they are to show a negative attitude toward accompanied driving.

Notably, the present study found that young drivers’ negative attitudes, such as disapproval, are positively correlated with their number of traffic violations and accidents while driving, though few participants had reported traffic violations (22.8%) or traffic accidents (6.2%) after their accompanied driving phase. The results show direct associations between young drivers’ attitudes toward accompanied driving and their traffic violations, which might help generate interventions targeted at accompanied driving. Although only those drivers who had accompanied driving experience were asked to participate and finish the scales in this study, the findings are very important and useful. As accompanied driving is getting more and more popular, the findings of this study provide evidence to make accompanied driving a compulsory step after young teens obtained their driving licenses in China.

There are limitations in this study. One limitation is that the associations between the ATADS-C factors and driving style might be susceptible to the effect of social desirability given that self-report methods were used. However, the main purpose of this study was to examine the reliability and validity of the newly-developed scale. Similar to previous studies [6], the self-report technique was felt to be the most suitable for understanding personal attitudes and behaviors. Another limitation is that few of the participants had been involved in traffic accidents due to having relatively less driving experience. A follow-up study with a larger sample is needed to further investigate the associations between the ATADS-C factors and traffic accidents.

In summary, the Chinese version of the ATADS has adequate psychometric properties and is a reliable and validated instrument in the Chinese driving context. This study is the first to reveal that young drivers’ attitudes toward accompanied driving, such as tension and disapproval, are associated with maladaptive driving styles. The findings provide evidence for the usefulness of the ATADS in young driver training and assessment in China.

## Supporting information

S1 File The ATADS in original language. (DOCX)

S2 File Attitudes Toward Accompanied Driving Scale -Chinese version. (DOC)
