# Supplementary material for: Psychometric properties of the Chinese version of the Attitudes Toward Accompanied Driving Scale and its relationship with driving styles
Source: PLoS One. 2020 Nov 19;15(11):e0242374. doi: 10.1371/journal.pone.0242374 (PMC7676715; doi:10.1371/journal.pone.0242374)
Supplement: S1 File — (DOCX) [file pone.0242374.s001.docx]

S1 File. The ATADS in original language

| ATADS-C items | Not at all |  | Sometimes |  | Very much |
| --- | --- | --- | --- | --- | --- |
| *Tension* | 1 | 2 | 3 | 4 | 5 |
| 3. I was usually a nervous wreck when I got out of the car after accompanied driving. | 1 | 2 | 3 | 4 | 5 |
| 7. Accompanied driving generated a lot of tension. | 1 | 2 | 3 | 4 | 5 |
| 4. I felt calm and at ease during accompanied driving. | 1 | 2 | 3 | 4 | 5 |
| 1. For us, accompanied driving is a battle | 1 | 2 | 3 | 4 | 5 |
| 6. I sometimes stopped, or almost stopped, the accompanied driving session because I was angry about something that happened during it. | 1 | 2 | 3 | 4 | 5 |
| 2. There were a lot of conflicts during our accompanied driving. | 1 | 2 | 3 | 4 | 5 |
| 5. I felt uncomfortable during, before, or after accompanied driving | 1 | 2 | 3 | 4 | 5 |
| *Disapproval* | 1 | 2 | 3 | 4 | 5 |
| 12. I often expressed criticism of the way my accompanying driver wanted me to drive or the way he/she was handling accompanied driving. | 1 | 2 | 3 | 4 | 5 |
| 11. Accompanied driving widened the gaps between me and my accompanying driver. | 1 | 2 | 3 | 4 | 5 |
| 10. During accompanied driving, I thought I had to take the lead, and sometimes even impose my will on my accompanying driver, for the process to succeed. | 1 | 2 | 3 | 4 | 5 |
| 13. My accompanying driver often expressed criticism of the way I was driving or the way I was handling accompanied driving. | 1 | 2 | 3 | 4 | 5 |
| *Anxiety* | 1 | 2 | 3 | 4 | 5 |
| 15. Most of the time I preferred to keep quiet during accompanied driving. | 1 | 2 | 3 | 4 | 5 |
| 17. I was apprehensive of the conflicts that might arise during accompanied driving. | 1 | 2 | 3 | 4 | 5 |
| 16. I was afraid that being with an accompanying driver would cause me to be involved in a traffic accident. | 1 | 2 | 3 | 4 | 5 |
| 14. I was afraid that I might make mistakes on the road because of the stress I felt during accompanied driving. | 1 | 2 | 3 | 4 | 5 |
| *Avoidance* | 1 | 2 | 3 | 4 | 5 |
| 20. On the whole, I wanted to spend as little time as possible behind the wheel during the accompanied driving period. | 1 | 2 | 3 | 4 | 5 |
| 19. Overall, I tended to avoid driving during the accompanied driving period. | 1 | 2 | 3 | 4 | 5 |
| 18. I tried to get in as many driving hours as possible during the accompanied driving period. | 1 | 2 | 3 | 4 | 5 |
| *Relatedness* |  |  |  |  |  |
| 9. Accompanied driving generated a sense of closeness between me and my accompanying driver. |  |  |  |  |  |
| 8. Accompanied driving gave me and my accompanying driver an opportunity to bond in a way we hadn’t done in a long time. |  |  |  |  |  |

Citation: Taubman-Ben-Ari O. Young drivers’ attitudes toward accompanied driving: A new multidimensional measure. Accid Anal Prev. 2010; 42(4):1009-1017. https://doi.org/10.1016/j.aap.2009.12.003
