# Supplementary material for: Psychometric properties of the Chinese version of the Attitudes Toward Accompanied Driving Scale and its relationship with driving styles
Source: PLoS One. 2020 Nov 19;15(11):e0242374. doi: 10.1371/journal.pone.0242374 (PMC7676715; doi:10.1371/journal.pone.0242374)
Supplement: S2 File — (DOC) [file pone.0242374.s002.doc]

S2 File. Attitudes Toward Accompanied Driving Scale -Chinese version

陪伴驾驶态度量表（中文版）

| 紧张 | 根本不 | 比较不 | 有时 | 经常 | 非常 |
| --- | --- | --- | --- | --- | --- |
| 3.当陪伴驾驶结束时，我常常会感到精神崩溃 | 1 | 2 | 3 | 4 | 5 |
| 7.陪伴驾驶产生了很大的紧张感 | 1 | 2 | 3 | 4 | 5 |
| 4.在陪伴驾驶期间，我感到平静和轻松 | 1 | 2 | 3 | 4 | 5 |
| 1.对我们来说，陪伴驾驶是一场战斗 | 1 | 2 | 3 | 4 | 5 |
| 6.我有时不太愿意接受陪同驾驶，因为我对陪伴驾驶期间发生的事情感到愤怒 | 1 | 2 | 3 | 4 | 5 |
| 2.在陪伴驾驶中有很多冲突 | 1 | 2 | 3 | 4 | 5 |
| 5.在陪伴驾驶期间、之前或之后，我感到不舒服 | 1 | 2 | 3 | 4 | 5 |
| 不赞成 | 1 | 2 | 3 | 4 | 5 |
| 12.我经常对我的陪同驾驶员希望我驾驶的方式，或者他/她处理陪同驾驶的方式提出批评 | 1 | 2 | 3 | 4 | 5 |
| 11.陪伴驾驶扩大了我和我的陪伴驾驶员之间的分歧 | 1 | 2 | 3 | 4 | 5 |
| 10.在陪伴驾驶过程中，我认为我必须带头，有时甚至把我的意愿强加给我的陪伴驾驶员 | 1 | 2 | 3 | 4 | 5 |
| 13.我的陪伴驾驶员经常对我的驾驶方式或我处理陪伴驾驶的方式提出批评 | 1 | 2 | 3 | 4 | 5 |
| 焦虑 | 1 | 2 | 3 | 4 | 5 |
| 15.大多数时候，我喜欢在陪伴驾驶时保持安静 | 1 | 2 | 3 | 4 | 5 |
| 17.我担心陪伴驾驶期间可能出现冲突 | 1 | 2 | 3 | 4 | 5 |
| 16.我担心和陪伴驾驶员在一起会导致我卷入了一起交通事故 | 1 | 2 | 3 | 4 | 5 |
| 14.我担心我会因为陪伴驾驶的压力而在道路上犯错误 | 1 | 2 | 3 | 4 | 5 |
| 避免 | 1 | 2 | 3 | 4 | 5 |
| 20.总体上，我想在陪伴驾驶期间尽可能不去开车 | 1 | 2 | 3 | 4 | 5 |
| 19.总的来说，我倾向于避免在陪伴驾驶期间开车 | 1 | 2 | 3 | 4 | 5 |
| 18.在陪伴驾驶期间，我尽量争取更多的驾驶时间 | 1 | 2 | 3 | 4 | 5 |
| 相互 | 1 | 2 | 3 | 4 | 5 |
| 9.陪伴驾驶在我和我的陪伴驾驶员之间产生了一种亲密感 | 1 | 2 | 3 | 4 | 5 |
| 8.陪伴驾驶给了我和我的陪伴驾驶员一个机会来加强彼此关系 | 1 | 2 | 3 | 4 | 5 |
